# Astrocytes: Dissecting Their Diverse Roles in Amyotrophic Lateral Sclerosis and Frontotemporal Dementia

**DOI:** 10.3390/cells12111450

**Published:** 2023-05-23

**Authors:** Chiara F. Valori, Claudia Sulmona, Liliana Brambilla, Daniela Rossi

**Affiliations:** 1 Molecular Neuropathology of Neurodegenerative Diseases, German Centre for Neurodegenerative Diseases (DZNE), 72072 Tübingen, Germany; 2 Department of Neuropathology, University of Tübingen, 72076 Tübingen, Germany; 3 Laboratory for Research on Neurodegenerative Disorders, Istituti Clinici Scientifici Maugeri IRCCS, 27100 Pavia, Italy

**Keywords:** astrocytes, ALS, FTD, animal models, cell models, rehabilitation, personalized medicine

## Abstract

Amyotrophic lateral sclerosis (ALS) and frontotemporal dementia (FTD) are fatal neurodegenerative disorders often co-occurring in the same patient, a feature that suggests a common origin of the two diseases. Consistently, pathological inclusions of the same proteins as well as mutations in the same genes can be identified in both ALS/FTD. Although many studies have described several disrupted pathways within neurons, glial cells are also regarded as crucial pathogenetic contributors in ALS/FTD. Here, we focus our attention on astrocytes, a heterogenous population of glial cells that perform several functions for optimal central nervous system homeostasis. Firstly, we discuss how post-mortem material from ALS/FTD patients supports astrocyte dysfunction around three pillars: neuroinflammation, abnormal protein aggregation, and atrophy/degeneration. Furthermore, we summarize current attempts at monitoring astrocyte functions in living patients using either novel imaging strategies or soluble biomarkers. We then address how astrocyte pathology is recapitulated in animal and cellular models of ALS/FTD and how we used these models both to understand the molecular mechanisms driving glial dysfunction and as platforms for pre-clinical testing of therapeutics. Finally, we present the current clinical trials for ALS/FTD, restricting our discussion to treatments that modulate astrocyte functions, directly or indirectly.

## 1. Introduction

Amyotrophic Lateral Sclerosis (ALS) is the most common form of adult-onset motoneuron disease, with an incidence of 1–2 cases/year/100,000 individuals. The disease presents with a wide variety of symptoms arising from motoneuron degeneration. The site of symptom onset normally provides a first criterion to stratify patients. More specifically, bulbar onset is declared when the disease appears with difficulties in swallowing (dysphagia) and articulating speech (dysarthria), while fasciculation, tremors, and muscular tone loss are hallmarks of spinal-onset ALS. In addition to these motor disabilities, about 15% of patients develop cognitive and behavioral impairments that call for an additional diagnosis of Frontotemporal Dementia (FTD). Patients can be further stratified on the basis of the etiological origin of the disorder. In 90–95% of occurrences, it appears sporadically (sALS), with many environmental and behavioral agents implicated in its pathogenesis, whereas it is inherited in the remaining 5–10% of instances, mainly as an autosomal dominant character (fALS). Mutations in several genes have been associated with the disease. From a neuropathological standpoint, post-mortem analysis reveals the loss of the motor cortex and spinal cord motor neurons as well as reactive gliosis. In addition, the cytoplasm of surviving neurons and glial cells shows abnormal proteinaceous aggregates, known as inclusions, whose defining element is the protein Transactive Response DNA-binding Protein 43 (TDP-43), which is present in 95% of cases (reviewed in [1]).

FTD is a clinical umbrella term that defines the second most prevalent form of dementia, grouping patients with different manifestations of behavioral, cognitive, and linguistic impairments. The underlying neuropathological condition is the degeneration of neurons in the frontal and temporal lobes (frontotemporal lobe degeneration, FTLD). Hereafter, we will, therefore, refer to FTD when discussing findings relative to the clinical condition, and FTLD will be reserved to evidence gathered at the biochemical, pathological, and cellular levels. Importantly, about 20% of FTD individuals develop motor dysfunction, meeting the criteria for an additional ALS diagnosis. The majority of cases cannot be ascribed to a known cause, while about 30% of occurrences are of a genetic origin, as mutations in different genes, including some linked to fALS, have been associated with the disease [2]. Intriguingly, the neuropathological assessment of FTLD cortical specimens also revealed the presence of inclusions either consisting of TDP-43 in about 45% of cases or comprising the cytoskeletal protein tau in another 45% of patients [2].

**Figure 1:**
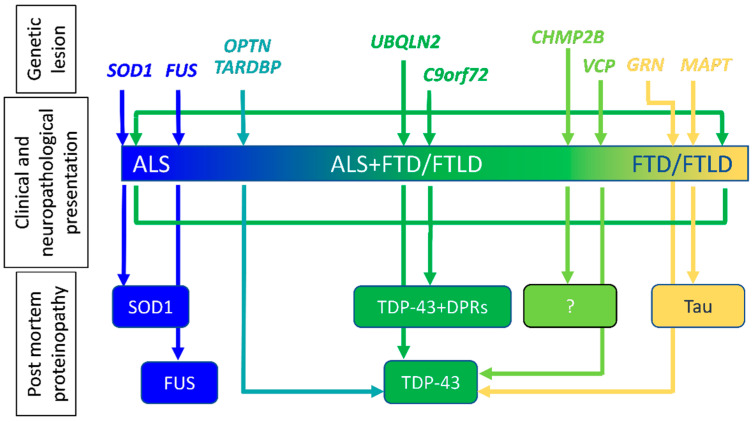
Familial ALS and FTD/FTLD: from genes to pathology. Several genes have been implicated in both ALS and FTD/FTLD. Curiously, some are equally associated with both disorders (i.e., *C9orf72*), while others (i.e., *SOD1* for ALS or *MAPT* for FTD/FTLD) predominantly cause one disease with a distinct neuropathological signature. Intriguingly, other genes (i.e., *TARDBP* for ALS or *GRN* for FTD/FTLD) are predominantly associated with different diseases, although they share the same pathological lesion (TDP-43 accumulation). *SOD1*, *superoxide dismutase 1*; *FUS*, *fused in sarcoma*; *OPTN*, *optineurin*; *TARDBP*, *TAR (transactive response) DNA Binding Protein*; *UBQLN2*, *Ubiquilin2*; *C9orf72*, *Chromosome 9 open reading frame 72*; *CHMP2B*, *Charged Multivesicular Body Protein 2B*; *VCP*, *Valosin Containing Protein*; *GRN*, *progranulin*; *MAPT*, *Microtubule Associated Protein Tau*; TDP-43, Transactive response DNA binding protein 43 kDa, the protein expressed from *TARDBP*. Blue: genes and proteins associated with a pure ALS phenotype. Yellow: genes and protein associated with FTD/FTLD. Green: genes and protein associated with the whole disease spectrum.

Altogether, this amount of evidence not only highlights the overlap between ALS and FTD/FTLD at the clinical, genetic, and biochemical levels (Figure 1), but also raises important questions, the answers to which will likely provide the basis for a deeper understanding of mechanisms driving those pathological conditions. This, in turn, may help establish suitable therapeutic interventions. Examples of key questions are: what are the mechanisms leading different gene mutations (e.g., *C9orf72* and *GRN*) to cause the same neuropathological lesion (TDP-43 inclusions), though with a different clinical manifestation (ALS and/or FTD for *C9orf72*, purely FTD for *GRN*); and can we extend mechanisms uncovered using models of a specific familial form of the disease (i.e., ALS-*SOD1*) to other genetic forms and sporadic cases?

On a mechanistic standpoint, the disruption of several pathways has been associated with ALS or FTLD, such as oxidative stress, excitotoxicity, aberrant RNA metabolism, and impaired protein homeostasis [3]. Remarkably, cellular dysfunction may not be limited to neurons, but could affect also glial cells. The non-cell autonomous contribution of the latter has been a particularly hot topic in the context of ALS research in the last few years (reviewed in [4]) and is now gaining momentum as a potentially contributing factor also in FTD (reviewed in [5]). Importantly, a recent analysis of the transcriptional profile of the frontal and the motor cortices of ALS cases suggested that patients could be classified into three molecular subtypes, respectively, characterized by (i) glial activation, (ii) oxidative stress and synaptic dysregulation, or (iii) transcriptional dysregulation [6]. Remarkably, the subset of patients showing a pronounced glial involvement displayed the faster progression [7].

Here, we focus our attention on astrocytes, one of the most abundant glial cell populations in the central nervous system (CNS). After briefly discussing elements of astrocyte physiology and their response to insults, we will recapitulate recent evidence showing how their functions are perturbed in ALS/FTD patients as well as in cellular and animal models of the disease. Finally, we will address how setting the focus on astrocytes could lead to the validation of informative biomarkers as well as provide valuable therapeutic options.

## 2. Astrocytes: From Physiology to Pathology

The recognition of astrocytes as an array of highly heterogenous cells was achieved by neuropathological analyses in the 19th century (reviewed in [8]). More recently, this hallmark was further corroborated, at the molecular level, by several single-cell or single-nuclei RNA sequencing studies (sc/snRNA-seq) as well as by advanced imaging techniques supporting the existence of functionally distinct astrocyte subpopulations [9]. A recent ground-breaking investigation not only correlated the expression of gene modules to distinct morphologies, but also mechanistically demonstrated that reducing the expression of key genes in astrocytes could both alter their morphology as well as affect neural circuit function in mice [10]. In the same study, it was discovered that genes belonging to modules that were crucial for astrocyte morphology were strictly associated with gene datasets related to several neurological conditions, including ALS [10].

Several important implications stem from this evidence. Firstly, it explains the extraordinary versatility of astrocytes, which are implicated in a wide array of homeostatic activities in the CNS, ranging from providing metabolic and trophic support to neurons and regulating the extracellular concentration of neurotransmitters and K^+^ ions to contributing to the protective functions of the blood–brain barrier (BBB) and the glymphatic system [11,12]. Secondly, it urges for an in-depth re-interpretation of the results obtained from animal models, in light of the growing number of differences acknowledged between rodent and human astrocytes [13,14]. Thirdly, it suggests that any findings observed in human inducible pluripotent stem cell (iPSC)-derived astrocytes should be regarded with keen awareness of the protocol used for differentiation, as there are now several methods available to generate region-specific cells [15]. On top of this, it is yet to be determined how faithfully those protocols allow for the recapitulation of the phenotype of distinct astrocyte subpopulations, by comparing their transcriptional profile with scRNA-seq studies.

Given such remarkable functional heterogeneity under physiological conditions, it is not surprising that astrocytes undergo substantial remodeling upon CNS insult, a phenomenon that has been known for decades as “reactive gliosis”, an umbrella term used to encompass complex morphological and transcriptional changes sustaining a phenotypic switch that promotes an inflammatory milieu. Such remodeling is highly regulated and leads to several transcriptional changes, some of which are shared by different insults, while others are tailor-made [16,17]. An early study of the astrocyte transcriptome proposed that the nature of the insult determines whether astrocytes can either be converted into neurotoxic cells or develop a neuroprotective phenotype in a dichotomic way [18,19]. However, this hypothesis has been recently rejected in favor of a more articulated view of the complexity of the astrocyte response (reviewed in [20]). More specifically, in the context of neurodegenerative disorders, it is of outstanding importance to discern astrocytes that retain homeostatic activities and/or boost a neuroprotective phenotype from cells that exacerbate a toxic environment. Recent evidence supports the co-existence of these glial populations in ALS tissues [7], information that appears crucial to develop a suitably targeted therapeutic strategy.

Furthermore, given the intimate interplay between astrocytes and the cells in their immediate neighborhood (neurons and other glial populations), we envision that our understanding of the true nature and extent of cell-to-cell astrocytic signaling may massively improve in the near future with the refinement of visual transcriptomics techniques, thereby allowing unbiased in situ investigations of gene expression.

## 3. Astrocytes as Active Players in the Pathogenesis of the ALS/FTLD Spectrum

### 3.1. Evidence from Patients

**Figure 2:**
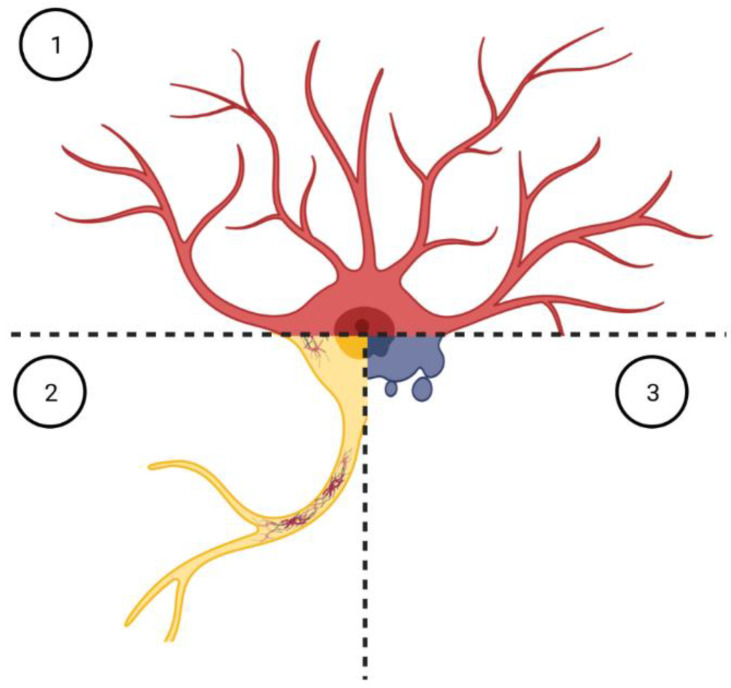
Facets of astrocyte pathology in ALS/FTLD. Neuropathological inspection of post-mortem material from ALS/FTLD patients revealed different aspects of astrocyte involvement, namely, inflammation (1), inclusion body formation (2), and atrophy/degeneration (3). Whether these phenotypes correspond to different moments along a continuous timeline or whether they rather affect distinct subpopulations of astrocytes deserve further investigations. Figure generated with BioRender.com.

Neuropathological analyses revealed three facets of astrocyte abnormalities in ALS/FTLD, namely, inflammation, the presence of protein inclusions, and atrophy/degeneration (Figure 2).

As recapitulated in the previous section, reactive gliosis is a well-recognized aspect of neuroinflammation that can be found in most neurodegenerative conditions, including ALS (reviewed in [21]) and FTLD [22]. In the spinal cord of ALS patients, Schiffer et al. [23] reported that reactive astrocytes display distinct morphologies and an increased density at the entering points of the corticospinal tract in the grey matter. At these points, no pathological changes affecting the entering fibers were observed; however, more prominent gliosis was found to occur in the lateral funiculus in proximity to a high number of dystrophic neurites. This finding was interpreted by the authors as indicative of the fact that corticospinal fibers follow a “dying back” pattern. Further morphological abnormalities of the astrocytes include reduced association of astrocytic processes with motor neuron somata [24] and with the mural basement membrane of the blood vessels [25].

From a neuropathological standpoint, the investigation on ALS tissues revealed that astrocytes bear hyaline [26], ubiquitin-positive [27,28,29,30] or, rarely, TDP-43-positive inclusions [31]. Furthermore, inclusions of the microtubule associated protein tau are abundant in astrocytes in various subtypes of frontotemporal lobar degeneration with tau protein pathology (FTLD-tau) (reviewed in [32]), a group of pathologies that does not present ALS co-morbidity, however (Figure 1). Nonetheless, phosphorylated TDP-43 inclusions were reported in astrocytes from a small cohort of patients affected by atypical FTLD-tau [33]. Finally, atypical astrocyte and neuronal tauopathy has been detected with starkly increased frequency in patients harboring the A/A major risk genotype at the single nucleotide polymorphism (SNP) rs1990622 on *TMEM106b* [34], a well-established genetic disease modifier in the ALS/FTD spectrum (reviewed in [35]).

The last aspect of astrocyte dysfunction to be described in ALS/FTLD is atrophy/degeneration, an event that has been localized both in the spinal cord of sALS patients [28] and in different cortical layers as well as at the interface between the grey and the white matter of FTLD patients, where astrocyte apoptosis was found to correlate with disease severity [36,37,38].

These neuropathological findings characterized astrocyte damage at a macroscopic level but did not provide insights as to how the pathological process affects the molecular signature of these cells. To acquire this type of information, recently developed techniques, such as snRNA-seq and spatial transcriptomics, have been applied to post-mortem tissues. In particular, a recent study, aiming at investigating the transcriptome of non-neuronal cells in FTD patients carrying *GRN* mutations (FTLD-*GRN)*, revealed the appearance of a disease-associated astrocyte subpopulation characterized by the expression of WDR49 (WD repeat domain 49) and the perturbation of several grey matter astrocyte pathways, including signaling to neurons, other parenchymal cells, and endothelial cells [39]. Intriguingly, another group recently reported astrocyte dysfunction in FTLD-*GRN*, with the upregulation of *GJA1* (coding for Gap junction alpha-1 protein, also known as connexin 43) and *AQP4* (coding for the water channel protein Aquaporin-4), thus suggesting aberrant coupling within the glial syncytium as well as with the blood vessels [40]. Complementary, a proteomic study, followed by expression-weighted cell type enrichment (EWCE), uncovered altered cellular metabolism in astrocytes in the grey matter of the frontal and temporal lobes of FTLD-*GRN* patients [41]. Although the overexpression of astrocyte-specific proteins, such as the glial fibrillary acidic protein (GFAP), was detected also in patients with frontotemporal lobar degeneration with *MAPT* mutation(s) (FTLD-*MAPT*), the EWCE analysis pointed towards functionally defective oligodendrocytes [41]. The involvement of distinct astrocyte subpopulations in FTLD-TDP vs. FTLD-tau was further corroborated by a study combining a post-mortem magnetic resonance imaging (MRI) investigation of the whole brain hemisphere with histological analysis [42]. Specifically, FTLD-tau patients displayed iron accumulation in astrocytes and microglia of the deep grey matter layer and in the white matter, while, in FTLD-TDP patients, iron-positive astrocytes were detected in the proximity of the blood vessels of the superficial cortical layers [42]. Although no peer-reviewed articles have been published to date (28 April 2023) on human post-mortem specimens from ALS patients, several publications describing snRNA-seq analyses have been recently deposited in a preprint repository [43,44,45,46,47]. Thus, we envision that we will soon gain new stimulating insights into astrocyte dysfunction in ALS/FTLD. In the meantime, spatial transcriptomics performed in a mouse model of the disease revealed the existence of gene modules associated with astrocytes displaying a distinct behavior along phenotype progression, thus further supporting the existence of different subpopulations [48]. Intriguingly, Maniatis et al. identified a correspondence between some gene expression modules in mice and those in the spinal cord of ALS patients [48]. In a small-scale study, spatial transcriptomics allowed for the identification of the dysregulation of *GRM3* (coding for Glutamate metabotropic receptor 3) and *USP47* (coding for Ubiquitin Specific Peptidase 47) genes in the cerebellum of an ALS-*C9orf72* patient, a finding which was corroborated in different brain areas of a larger cohort of patients, including ALS-*C9orf72*, ALS-*SOD1,* and sALS cases, using an in situ hybridization technique [49]. However, one issue that should be carefully considered is the insufficient resolution of published spatial transcriptomics studies, as they combine the RNA extracted from 100 µm-diameter areas into a single spot, thereby precluding the possibility of pinpointing the exact cell type displaying differential gene expression. This is a critical aspect since *GRM3* is expressed by a wide variety of cells in addition to astrocytes. We envision that the refinement of spatial transcriptomics to achieve single-cell resolution will clarify such ambiguities in the near future. Taken together, this amount of evidence implies that the astrocytic response against the neurodegenerative process occurring in FTLD is two-fold: on the one hand, it presents multifaceted features, combining neuroprotective with detrimental aspects; on the other hand, it is tailored to the specific type of genetic or biochemical lesion that triggers the disease.

Previous evidence supported the hypothesis that astrocytes play a complex role in the pathogenesis of ALS and FTLD. A notable limitation of these findings is that they were recorded in post-mortem material, thereby providing a snapshot of the final stage of astrocyte dysfunction. To overcome this drawback and gain information as to how glial pathology evolves, there is an urgent need to monitor astrocytes during disease progression. The development of astrocyte-specific ligands, such as [11C](L)-deprenyl-D2, which enables the measurement of monoamine oxidase B (MAO-B) levels, has indeed served as a powerful tool to track astrocyte activation by positron emission tomography (PET) in ALS [50]. Complementary, tools to monitor the activity of distinct brain structures relying on astrocyte function, such as the glymphatic system or the BBB, could provide information as to whether astrocytes undergo a specific functional deficit along disease progression. To this end, the development of a novel neuroimaging method, known as DTI-ALPS (diffusion tensor image analysis along the perivascular space), has enabled the ability to monitor the efficiency of the glymphatic system [51]. This method has been rapidly implemented in neuroimaging facilities, thereby providing insights into an astrocyte-specific dysfunction in living patients affected by several neurological conditions, including Alzheimer’s disease [52] and Parkinson’s disease [53]. We anticipate that similar studies will be soon performed on cohorts of ALS and FTD patients as well. The activity of the glymphatic system could also be inferred by administering the gadolinium-based MRI-contrast agent gadobutrol, by allowing its penetration in precisely selected areas of the brain parenchyma by magnetic resonance–guided focused ultrasound surgery (MRgFUS), and, finally, by monitoring its dynamics by contrast-fluid-attenuated inversion recovery [54]. Notably, a case-report study presented the results obtained from an ALS patient using MRgFUS, thereby supporting the feasibility of this technique in the context of motor neuron disorders [54].

A complementary approach to monitor astrocyte impairment along disease progression would be to identify and validate soluble biomarkers that can report such dysfunction. For example, assessing the BBB integrity may serve as a proxy to monitor astrocyte activity since these cells play a crucial role in the formation and maintenance of the barrier [55]. Interestingly, dysfunction of the BBB has been long recognized in human ALS cases [56,57] and, more recently, its increased permeability has been associated with a worse prognosis and seems to be more pronounced in patients affected by ALS/FTD than in those with ALS only [58]. In agreement with this observation, another recent study confirmed the association between enhanced BBB impairment and increased risk of death in ALS patients with spinal onset [59]. However, in a larger study aiming at evaluating BBB dysfunction in patients with different forms of dementia, no association could be found with FTD patients [60].

These conflicting results urged for the identification and validation of alternative astrocyte-specific biomarkers, as BBB dysfunction may also arise from impaired functionality of other cellular partners contributing to its homeostasis. To reach this goal, several studies were performed to investigate the levels of proteins, typically expressed by activated astrocytes, in the cerebrospinal fluid of patients affected by different neurodegenerative diseases. Interestingly, chitinase-3-like protein 1 (CHI3L1, or YKL-40) expression was reported to discriminate patients who received a double ALS/FTD diagnosis from those with FTD with no impairment of motor capabilities [61]. Moreover, significantly elevated levels of CHI3L1 have been demonstrated in other studies [62,63], thus suggesting that monitoring astrocyte dysfunction could be a useful tool not only to monitor disease progression but possibly also for differential diagnosis. Interestingly, CHI3L1 appears to be specifically expressed by a subpopulation of white matter astrocytes [62], thereby further reinforcing the hypothesis that different astrocyte populations provide distinct contributions to disease pathogenesis.

Another glial protein, which is currently under investigation as a potential biomarker, is the cytoskeletal protein GFAP. In serum, its concentration is higher in ALS patients with cognitive impairment [64] and negatively correlates with cognitive scores [65]. Furthermore, the abundance of GFAP in the plasma of FTD patients, alone or in combination with the neurofilament light chain protein, revealed that the simultaneous assessment of these two proteins could serve as a differential diagnosis biomarker assay to discriminate between FTD and Alzheimer disease patients [66] as well as between molecularly distinct FTLD subtypes [67]. Furthermore, plasma GFAP could act as a progression biomarker as its level correlates with a higher burden of cognitive impairment during FTD disease progression [66]. In the future, we envision that other molecules of astrocyte origin might be explored as potential biomarkers, such as specific microRNAs [68].

These biomarker studies point towards the intriguing possibility that the dysfunction of astrocyte subpopulations could be the ultimate disease modifier, aggravating neurodegeneration in different CNS regions and, in turn, leading to the development of more complex disease manifestations. Further indirect evidence supports this hypothesis: the composition of the gut microbiota, which has an important tuning role on astrocytes homeostasis [69,70,71,72], also modulates disease progression in ALS patients (reviewed in [73]). In addition, a recent study demonstrated that gut dysbiosis worsens disease course in patients with spinal-onset, while oral dysbiosis negatively impacts patients with bulbar onset of ALS [74].

Although no published studies are currently available that explore the gut microbiota in FTD cases, we can speculate that similar findings may be extended to those patients in light of the recent finding showing that the ALS/FTD-associated *C9orf72* gene plays a role in subduing a pathological inflammatory response triggered by gut bacteria [75].

### 3.2. Evidence from Animal Models

The observations presented in the previous section indisputably support the notion that several intertwined mechanisms are likely to contribute to the pathogenesis of the ALS/FTLD spectrum. It is, therefore, imperative to develop animal and cellular models that allow for the dissection of the different mechanistic aspects and, thus, support the development of suitable therapies. The ideal animal model should recapitulate every aspect of the disease: the phenotype should be similar to clinical symptoms and should correlate with analogous molecular and neuropathological abnormalities, including astrocyte dysfunction. A comprehensive discussion on all the available animal models of ALS/FTLD goes beyond the scope of this review, and can be appreciated elsewhere [76]. Here, we summarize evidence supporting the role of astrocytes in ALS/FTLD animal models.

In 1993, the groundbreaking discovery of *Superoxide Dismutase 1* (*SOD1*), as the first gene associated with fALS [77], led to the generation of several transgenic mouse strains expressing various mutant versions of its translated product, the Cu/Zn-SOD (SOD1) enzyme. Importantly, the phenotype of these animals closely resembles human ALS, as they develop tremors and muscular weakness, rapidly progressing into paralysis and, ultimately, death. From the histopathological standpoint, they also recapitulate many aspects of the human condition, including severe motoneuron loss, ubiquitin-positive inclusions, and microgliosis. In terms of astrocyte dysfunction, reactive gliosis, inclusions, and cell demise have been described. Intriguingly, in one such transgenic mouse line, the earliest detected abnormality was the presence of SOD1- and ubiquitin-positive inclusions in astrocytes, thus suggesting that these cells are directly impaired by mutant SOD1 expression, and, consequently, motoneuron loss could be a consequence of glial dysfunction [27]. Selective transgene-driven mutant SOD1 expression in astrocytes allowed for a test of this hypothesis. An early report demonstrated that SOD1^G86R^ (the murine orthologue of the fALS-associated hSOD1^G85R^) expression in GFAP-positive astrocytes is sufficient to trigger reactive gliosis but fails to trigger motoneuron demise [78]. Yet, several subsequent studies depicted a different scenario. Firstly, wild-type motoneurons surrounded by mutant SOD1-expressing glial cells displayed ubiquitin-positive inclusions in chimeric mice [79]. Furthermore, transplanting entopic mouse [80] or human astrocytes [81] that express mutant SOD1 into wild-type rodents instigated motor dysfunction and motoneuron degeneration. These studies consistently support the hypothesis that astrocytes expressing ALS-linked SOD1 mutants develop a neurotoxic phenotype. One may, therefore, argue that this feature is a hallmark of a distinct fALS subtype. In addition, transgenic rats expressing hTDP-43^M337V^ under the control of an inducible, astrocyte-specific promoter rapidly developed motor impairment, motoneuron loss, and ubiquitin-positive inclusions in astrocytes as well as astrogliosis and microgliosis [82]. Similarly, the expression of another fALS-associated mutant hTDP-43 in glial cells in the fruit flies resulted in neuromuscular junction impairment [83]. From a mechanistic standpoint, it is believed that inclusion body formation leads to loss of TDP-43 physiological functions, which, in turn, contribute to the pathogenesis of ALS/FTLD. In keeping with this, when TDP-43 expression was reduced predominantly in astrocytes by RNA interference, transgenic mice showed motor impairment progressing into paralysis, aberrant electromyography parameters, and motoneuron loss [84]. Analogously, astrocytes from transgenic mice having TDP-43 depleted from GFAP-positive cells display altered morphology and acquire a reactive transcriptome pattern [85]. This finding was intriguingly echoed by the recent discovery of the association between astrocyte morphology-defining genes and genes associated with ALS [10]. Furthermore, astrocyte-specific TDP-43 depletion was reported to trigger motor impairment in the absence of motoneuron loss in transgenic mice [85]. Finally, targeted overexpression of mutant FUS in spinal cord astrocytes by viral infection was sufficient to induce a neuroinflammatory phenotype, motoneuron loss, and motor impairment in mice [86].

Taken together, this amount of evidence consistently supports the notion that ALS/FTLD-associated proteins can influence the homeostasis of astrocytes, often turning their phenotype into a neurotoxic one. This conclusion has profound repercussions in terms of therapy development, as it hints that targeting astrocytes might be a valuable option to slow down or even halt neurodegeneration. To support the validity of this hypothesis, one should first ask whether healthy astrocytes preserve their ability to support their surrounding neurons during disease unfolding. In line with this, transplanting wild-type rat glial-restricted precursors [87], human iPSCs [88], or embryonic stem-cell-derived [89] astrocyte-committed glial precursors were shown to ameliorate the phenotype of ALS animal models. Furthermore, more recent studies demonstrated that astrocytes retained their neuroprotective abilities when co-cultured with motoneurons previously exposed to TDP-43 aggregates [90]. Finally, healthy astrocytes do not acquire a neurotoxic phenotype when exposed to the dipeptide repeat protein polyGA [91], a by-product of the hexanucleotide repeat expansion of the *C9orf72* gene [92].

A complementary approach may be based on reversing potentially aberrant pathways taking place cell-autonomously in astrocytes or affecting their communication with surrounding neuronal and glial partners. In this specific case, the mandatory prerequisite is to identify the de-regulated mechanisms. The next section reviews several studies that have significantly contributed to shed light into this issue.

## 4. Cell-Autonomous Mechanisms of Astrocyte Dysfunction in ALS/FTLD

As discussed in the previous sections, neuropathological assessment of astrocyte response to the ALS/FTLD spectrum can be summarized into three major events: inflammation, protein aggregation, and atrophy/cell death. As inflammation implicates the release of soluble mediators, and the latter can act in a paracrine way, this process cannot be included among cell-autonomous occurrences. Thus, we will address it in the next section, in the context of the aberrant interactions between astrocytes and other cells. Another important point to bear in mind is that these three phenomena might occur in parallel, or in different subpopulations of astrocytes, or they might rather capture different stages of a continuous detrimental process. In support of the latter possibility, we analyzed the spinal cord of hSOD1^G93A^ transgenic mice and found out that ubiquitin-positive morphologically aberrant astrocytes are degenerating cells, as they express the apoptotic marker active caspase-3 [28,93]. Further analyses of the same transgenic model revealed that activated astrocytes overexpress the BH3-interacting domain death agonist and other members of the BH3 protein family, which are well-known for their role in the signaling cascade driving mitochondrial stress into apoptosis. However, the absence of other markers of cell demise suggests that these studies have captured cells in an early stage of the detrimental process. Related to this point, it is important to remark that increased astrocyte vulnerability is not restricted to ALS-*SOD1* models but was confirmed also in differentiated patient-derived iPSCs carrying mutations in the *TARDBP* [94] or *VCP* [95] genes. Intriguingly, human astrocytes derived from fibroblasts of patients carrying *C9orf72* hexanucleotide repeat expansions displayed increased vulnerability to metabolic stressors [96,97]. However, astrocytes differentiated from a healthy donor and exposed to TDP-43 high molecular weight species neither underwent apoptosis nor mounted a pro-inflammatory phenotype [90], thus suggesting that the presence of aberrant TDP-43 in astrocytes was not sufficient to trigger the development of a detrimental phenotype. More recently, evidence of abnormal cell morphology and impaired proteostasis has been independently detected in iPSCs-derived astrocytes carrying mutations in the *MAPT* [98,99]. Moreover, defective autophagy, leading to endoplasmic reticulum stress in astrocytes, has been recognized as an early event in organoid slice cultures generated from patients harboring hexanucleotide repeat expansions in the *C9orf72* gene, which is also implicated in the pathogenesis of ALS and FTD [100]. Curiously, another group investigated astrocytes derived from a patient carrying a mutation in the gene *CHAMP2B* and uncovered enhanced autophagy [101]. This discrepancy could be explained by several differences between the two studies, such as the use of different models (i.e., 2D vs. 3D cultures), the difference in the genetics (i.e., *CHAMP2B* vs. *C9orf72*), and the biochemical lesions (i.e., yet unknown ubiquitinated protein vs. TDP-43). However, a more intriguing possibility is that astrocytes rely on a tightly controlled autophagy flux to maintain their homeostasis and any disturbance, whether it goes towards hyper- or hypo-activation, is detrimental. Thus, this calls for further investigations aiming at comprehending the homeostasis of specific proteins in astrocytes.

Parallel to atrophic astrocytes, a subpopulation of hyperproliferating cells was identified in both mouse [102,103] and rat [104] models of ALS-*SOD1*. However, the relevance of these cells to disease progression was likely limited, as their depletion was shown not to prolong the lifespan of transgenic ALS mice [103]. On the contrary, astrocyte apoptosis is likely to be a significant contributor to ALS pathogenesis, as the number of degenerating astrocytes ramps up during disease progression [28,93], and their accumulation correlates with earlier death in experimental mice [105]. Furthermore, pharmacological manipulations halting astrocyte apoptosis prolong survival in transgenic mice [28,93].

Based on these findings, it is of outstanding importance to understand the molecular mechanisms leading to astrocyte cell death. To this end, our group speculated that hSOD1^G93A^ astrocytes might develop enhanced vulnerability to glutamate toxicity since these cells are contiguous to glutamatergic synapses [28,93]. We established that not only are ALS astrocytes prone to succumb when challenged with this neurotransmitter, but, also, the deadly cascade is triggered by the activation of metabotropic glutamate receptor 5 (mGluR5) and progresses through aberrant Ca^2+^ signaling [28,93]. Importantly, impaired Ca^2+^ homeostasis has been independently identified by several groups and attributed either to the aberrant release of Ca^2+^ from the intracellular endoplasmic reticulum stores [28,106,107,108,109,110] or to excessive import of this ion from the extracellular environment [111]. It has also been recently demonstrated that aberrant Ca^2+^ signaling leads to impaired proteostasis and mitochondrial dysfunction (reviewed in [112]), thus potentially fueling detrimental vicious cycles within astrocytes. In agreement with this, iPSC-derived astrocytes modeling FTLD-tau displayed increased vulnerability to the mitochondrial poison rotenone, a well-known molecule inducing oxidative stress [98]. Moreover, human astrocytes carrying *CHAMP2B* mutations displayed aberrant mitochondrial morphologies and functions, thus leading to enhanced oxidative stress [101].

Taken together, these findings suggest that the phenotype of astrocytes undergoes substantial changes in the context of ALS and FTLD neurodegeneration, therefore, encouraging a comprehensive understanding of the pathways involved. Many groups independently applied unbiased analyses of the transcriptome of mouse astrocytes along disease progression [113,114,115,116] as well as of human astrocytes in monolayer cultures [117,118], human iPSC-derived cerebral organoids [119,120] or human post-mortem material [39]. Interestingly, a meta-analysis of these gene expression studies has been recently released [121] and showed that fALS astrocytes undertake a pro-inflammatory phenotype that shares similarities with the neurotoxic phenotype induced by a cytokine cocktail and originally named “A1” [19]. The relevance for disease progression of such a harmful phenotypic switch has been demonstrated in an ALS mouse model, where genetic manipulations were introduced to abrogate the expression of the pro-inflammatory factors driving the transition towards the “A1” status. Noteworthy, this study revealed that abolishing the key pro-inflammatory elements prolonged mouse survival [122]. However, the “A1” transcriptome fingerprint does not comprehensively describe the phenotype of fALS astrocytes and shares little overlap with the transcriptome changes displayed by sALS patients [121]. Further evidence that the “A1” status does not fully recapitulate the features of activated astrocytes even in fALS is provided by the proteomic analysis of conditioned medium harvested from astrocytes expressing hSOD1^G93A^ [123]. Finally, astrocytes carrying mutations in different genes associated with fALS (i.e., *VCP* and *SOD1*) mount a transcriptional response when they are treated with the “A1” inducing pro-inflammatory cytokine cocktail. This response not only is distinct among the different genotypes, but it also contains elements of both “A1” and “A2” statuses [118].

Altogether, these studies reinforce the idea that others have already emphasized, i.e., the requirement for a more precise description of the reactive astrocyte response [20]. In this regard, a recent elegant functional genomic study combined CRISPR-mediated gene ablation with scRNA-seq and computational analysis. The authors discovered that human iPSC-derived astrocytes responded to the “A1” induction protocol primarily by activating the transcription factor NF-kB. Then, they could diverge towards two distinct inflammatory states: the first one was driven by the transcription factor STAT3 and fueled by IL-6 signaling; the second one was regulated by interferon signaling [124]. Although not yet investigated in the context of ALS/FTLD research, these findings were particularly intriguing since STAT3 may also drive a neuroprotective phenotype when triggered by ephrin B1 signaling, a pathway activated by injured neurons, but was impaired in an ALS mouse model [125]. Furthermore, STAT3 signaling has been recently linked to astrocyte-mediated BBB dysfunction in different neurological conditions [126]. Finally, in cerebral organoids modeling FTLD-*MAPT*, scRNA-seq revealed increased IL-6 signaling in astrocytes [119]. Another example of the complexity of astrocyte reactivity comes from the analysis of the dynamics of the protein megalencephalic leukoencephalopathy with subcortical cysts 1. Under physiological conditions, it is considered a marker of perivascular astrocytes [127], but it is consistently overexpressed in astrocytic cell models of ALS [121] as well as in ALS patients [128]. Interestingly, this protein might play a role in self-limiting the inflammatory response [129] and might also counteract connexin 43 impairment in ALS [107] since it stabilizes connexin 43-containing gap junctions [130].

Complementary, a spatial transcriptomics study performed in the spinal cord of the hSOD1^G93A^ mouse model of fALS uncovered the existence of several astrocyte-related expression modules with distinct expression trajectories along disease progression [48]. Overall, these studies gave us valuable insights into the exquisite complexity of astrocyte inflammatory response to CNS insult in the context of ALS/FTD neurodegeneration.

Another relevant facet of astrocyte response in the ALS/FTLD disease spectrum emerging from several unbiased studies is the downregulation of various branches of their metabolism [97,101,131,132]. When looking into specific metabolites, deficient lactate synthesis and release was consistently reported in several astrocyte models [96,133,134]. Furthermore, hSOD1^G93A^ mouse astrocytes were shown to display deficient cholesterol biosynthesis [113], a finding that was recently extended to female hTDP-43^A315T^ mice [135] and, more importantly, to human cerebral organoids cultivated from FTLD-tau patients [120].

## 5. Non-Cell Autonomous Mechanisms of Astrocyte Dysfunction in ALS/FTLD

The ever-growing body of evidence addressed in the previous section suggests that the pathological milieu occurring in ALS/FTLD directly disrupts the homeostasis of astrocytes. Consequently, they may interact with their surrounding cell partners in new and aberrant ways. Here, we will specifically discuss how their interplay with neurons, other glial cells, and the BBB becomes dysfunctional in diseased conditions, specifically in that of ALS and FTD.

### 5.1. Neurons

As previously described, the expression of ALS/FTLD proteins in astrocytes is sufficient to trigger neurodegeneration in several animal models [79,80,81,82,84]. These findings strongly argue that astrocytes can become inherently toxic to neurons, a hypothesis that has been further corroborated by several independent co-culture studies of mouse [40,133,136,137,138,139,140,141,142] and human cells [40,142,143,144,145,146,147,148,149,150]. Several observations indicate that astrocyte toxicity is mediated by soluble factors [123,139,150,151,152,153,154], although no consensus has been achieved as to which molecule(s) is (are) the culprit(s). Early studies pointed at excessive extracellular glutamate due to reduced clearance by astrocytes, oxidative stress species, growth factors, and neuroinflammatory mediators as major neurodamaging agents (reviewed in [21] and further corroborated in more recent studies, such as [142,155,156]). However, in sharp contrast with the aforementioned evidence, a recent study investigating glutamate metabolism in astrocytes expressing FTD-associated *CHMP2B* variants unexpectedly uncovered enhanced uptake and metabolism of the neurotransmitter [157]. The authors suggested that the increased metabolism was due to an augmented expression in astrocytes of glutamine synthetase, the rate limiting enzyme converting glutamate to glutamine. Interestingly, they did not clarify whether this was specific to *CHMP2B* astrocytes, or if it may also be present in other FTD-associated mutant astrocytes. They further proposed that enhanced glutamate uptake represented an attempt of the astrocytes to remove glutamate that was released in excess by neurons, which failed to incorporate it in the tricarboxylic acid cycle due to reduced glutamate dehydrogenase 2 (GDH2) levels [157]. Whether the reduction in GDH2 expression is specific for neurons carrying *CHMP2B* mutations or is a general feature of this cell type in all forms of FTD deserves further investigations.

The discovery that ALS/FTD astrocytes display metabolic impairment led to the hypothesis that motor neuron toxicity could arise from a lack of adequate sustenance. In agreement with this vision, two strategies proved to be informative. As mouse ALS astrocytes display defective lactate release, a supplementation of mouse ALS astrocyte-motor neuron co-culture medium with this metabolite was first shown to rescue neuronal demise [133]. A similar result was achieved also by pre-treating human *C9orf72* hexanucleotide repeat expansions (HRE) astrocytes with inosine, which boosts their metabolism and, in turn, increases the synthesis and release of lactate [96]. Consistent with this finding, a recent study revealed that the PET tracer (SP-4-2)-[[2,2′-(1,2-dimethyl-1,2-ethanediylidene)bis[N-methylhydrazinecarbothioamidato-κN^2^,κS]](2-)]-copper (CuATSM) reduced elevated basal and ATP-linked mitochondrial respiration and increased glycolysis, leading to enhanced lactate release in human astrocytes derived from sALS patients or fALS cases carrying different genetic lesions. However, the molecular mechanisms underlying these events have not been fully elucidated and require further attention [158]. Interestingly, both studies involving human cells uncovered that, in spite of being consistently toxic to motoneurons, astrocytes displayed an unexpected patient-specific heterogeneous response to the treatment, thereby advocating for urgency of developing personalized medicine for the cure of ALS [96,158].

More recently, microRNAs were identified as another class of potential mediators of astrocyte-mediated neurotoxicity. In particular, mouse astrocytes expressing hSOD1^G93A^ downregulate miRNA146a. Restoring its expression in astrocytes reverted their neurotoxic phenotype [110,159]. Intriguingly, human astrocytes transdifferentiated from sALS, and fALS fibroblasts also displayed dysregulated miRNA146a expression and secretion. However, depending on the patient, the expression of miRNA146a could be either reduced or enhanced. Restoration of miRNA146a levels in depleted ALS astrocytes counteracted their reactive/inflammatory phenotype [160]. As re-establishing miRNA146a expression in astrocytes protected motoneuron-like cells in co-cultures, it is reasonable to postulate that any study addressing the therapeutic value of agents increasing its levels should be focused on patients displaying such deregulation [160]. Noteworthy, some astrocyte lines were found to exhibit miRNA146a upregulation and to trigger neurodegeneration in co-culture systems. However, the impact on motor neuron survival of decreasing its levels was not elucidated. Intriguingly, miRNA146a modulates the expression of a number of genes, such as the TNFR-associated factor 6 and the IL-1R-associated kinase [159], both inducers of the NF-kB inflammatory pathway [161].

Another astrocyte-derived microRNA of interest is miR-494-3p. This small RNA is released by astrocytes in the exosomes, and its expression is reduced in cells differentiated from patients carrying *C9orf72* HRE as well as in the spinal cord of sALS cases [162]. Restoring miR-494-3p levels induced the expression of its target, Semaphorin 3A, a critical gene for the maintenance of motor neuronal axons, and, importantly, rescued motor neuron demise in co-culture systems [162]. Furthermore, it is possible that other mechanisms might contribute to the neuroprotective effects of miR-494-3p restoration. For instance, miR-494-3p administration might also reduce the expression of its target gene protein phosphatase and tensin homolog (*PTEN*) [163], an event that sustains ALS-motoneuron survival [164] and mitigates NF-kB-mediated inflammation [165]. It is, therefore, tempting to speculate that these non-coding RNAs play a pivotal role in tuning inflammation in the context of ALS/FTLD.

Taken together, these studies suggest that the toxic interplay between astrocytes and neurons in ALS/ FTLD is likely mediated by a combination of several different aberrant cellular pathways in both cell types. This consideration implies that efforts should be made to prioritize the most robust candidates in the quest to identify the toxic element of the deadly astrocyte-neuron interplay in ALS. To this end, Mishra et al. [123] took an interesting approach by first performing a proteomic analysis of the conditioned medium from hSOD1^G93A^ expressing astrocytes. The results were then submitted to a novel bioinformatics analysis along with pre-existing data describing the transcriptome of motoneurons in order to identify and prioritize noxious ligand-receptor interactions. This strategy led to the discovery of aberrant signaling originating from the release of the amyloid precursor protein (APP) from astrocytes and the activation of the death receptor-6 (DR6) on motoneurons [123]. Importantly, the relevance of this finding was demonstrated by showing that the motoneuron demise, upon exposure to the ALS-astrocyte conditioned medium, can be prevented by either blocking APP release from astrocytes prior to producing the conditioned medium, or ablating the expression of DR6 on motoneurons [123].

### 5.2. Other Glial Cells

The cross-talk between different populations of glial cells has received less attention than the cell–cell communication signaling occurring between astrocytes and neurons. The characterization of transgenic animals with selective TDP-43 depletion from astrocytes, however, led to the discovery that astrocytes themselves acquire a neuroinflammatory phenotype, and microglia increase their reactivity markers [85]. These mice did not undergo motoneuron loss, but displayed a selective reduction in the number of mature oligodendrocytes, while the abundance and proliferation of oligodendrocyte precursors remained unaffected, a finding that may explain the motor deficits that these mice experience [85]. Intriguingly, the administration of an inflammatory astrocyte-conditioned medium to mature oligodendrocytes induced cell death in vitro, thus suggesting that mature oligodendrocyte degeneration could be a general response to an inflammatory microenvironment [166]. This observation supports the hypothesis that also oligodendrocytes become dysfunctional in ALS/FTLD, a topic which has recently gained more and more attention [167,168,169]. Finally, astrocyte-mediated microglial activation was also independently observed in a cell model of ALS-*SOD1*. More specifically, a conditioned medium from ALS-astrocytes was reported to induce a robust inflammatory response in a microglia-like cell line [110]. This microglial activation did not occur when miRNA-146a expression was restored in ALS astrocytes, thereby suggesting that it was linked to reduced expression of this microRNA [110]. This evidence, together with the aforementioned observation that restoring miRNA-146a in ALS astrocytes reverts their neurotoxic phenotype, strongly argues that miRNA-146a regulates a variety of pivotal targets to sustain astrocyte homeostatic properties.

### 5.3. BBB

The BBB and the blood–spinal cord barrier are the structures that tightly regulate the exchange of substances between the bloodstream and the CNS parenchyma. Perivascular astrocytes locate their endfeet around the capillaries and establish functional interactions with components of the BBB. They regulate its functions by exchanging signals with the other cell constituents, namely, endothelial cells and pericytes [127]. Notably, BBB leakage has been long recognized in ALS patients [56,57,170] as well as in several cellular and animal models of the disease [57,171,172,173,174,175,176,177]. It has been linked to endothelial cells, where reduced expression of proteins regulating their junctions might impair the tightness of their coupling [57,176]. In addition, endothelial cells in ALS patients [178,179] as well as in different experimental models of ALS [179] upregulate transporter proteins, such as P-glycoprotein, thereby suggesting an incorrect balancing between influx and extrusion of substances from the brain and spinal cord parenchyma. Intriguingly, such endothelial dysfunction is secondary to signaling arising from astrocytes, and several mechanisms have been proposed to drive this event. These include excessive release of glutamate [179], reactive oxidative species, and the pro-inflammatory cytokine TNFα [126,180]. BBB dysfunction has been comparatively less investigated in the context of FTLD. However, a recent snRNA-seq study performed on several cortical areas of FTD patients with *GRN* mutations suggested that diseased astrocytes display enhanced chemokine (C-X-C motif) ligand 1 signaling towards vascular cells, a cascade known to be associated with augmented BBB permeability [39]. Finally, additional strain on the BBB tightness could come from the overexpression [40,181,182,183,184,185] and/or mislocalization of the protein AQP4, a molecule of outstanding importance for the regulation of brain water homeostasis [186].

## 6. Astrocytes as Therapeutic Targets in ALS/FTLD

Several lines of evidence indicate that astrocyte dysfunction is not only a consistent feature of the ALS/FTLD spectrum but also contributes to the establishment of a neurotoxic environment through aberrant interactions with neurons and other neural cell types. Thus, astrocytes may represent an attractive target for therapeutic intervention in ALS/FTD. The previously mentioned transplantation experiments in animal models [87,88,89] already provide proof-of-principle evidence that restoring astrocyte function could be beneficial in ALS. Notably, the neuroprotective potential of transplanted glial precursor cells could be further boosted by engineering them to overexpress the glial cell line-derived neurotrophic factor (GDNF, [187]), a pro-survival protein that astrocytes tend to upregulate during the late-stages of disease progression in a mouse model of ALS and sALS patients [188]. Notably, blocking the signaling cascade that mediates GDNF upregulation in astrocytes was reported to lead to faster motor neuron loss and reduction in the lifespan of hSOD1^G93A^ mice [188].

Neural progenitor cells engineered to differentiate to astrocytes and to secrete GDNF (CNS10-NPC-GDNF) were produced under good manufacturing practice for a phase I/IIa clinical trial (NCT02943850) to test the safety of unilateral spinal cord injection in a small cohort of ALS patients [189]. The study met the primary end-point for safety but showed only a small delay in motor function loss in the treated side, most likely due to an unanticipated minimal migration of the injected cells from the dorsal towards the lumbar spinal cord [189]. These overall encouraging results will be hopefully reinforced by the results of an analogous phase I/IIa trial from an independent ALS clinic (NCT03482050) and by an ongoing study designed to evaluate the injection of CNS10-NPC-GDNF in the motor cortex of ALS patients (NCT05306457), thus possibly corroborating the therapeutic neuroprotective power of healthy astrocytes.

A complementary approach would be to identify a key molecule/pathway within astrocytes in order to design a suitable therapeutic strategy to be validated in animal models. In keeping with this strategy, an evident candidate was recognized in mutant SOD1s. Consistently, early genetic manipulations [190,191] or viral-mediated gene therapy [192,193] demonstrated that reducing the expression of this harmful protein in astrocytes slowed motor decline and prolonged survival in mouse models of ALS-*SOD1*. The success of these and several other studies aimed at silencing *SOD1* in various CNS populations (reviewed in [194]) led to testing the therapeutic potential of SOD1 ablation in ALS-*SOD1* patients. Regrettably, although a phase I/IIa clinical trial showed that the administration of an antisense oligonucleotide to lower *SOD1* expression in ALS patients was safe [195], a subsequent study very recently failed to demonstrate that the intervention slowed disease progression [196]. Notably, at the moment of publication of this latter study, most of the patients enrolled in this trial were still alive and participating in a long-term follow-up investigation, thereby leaving hope that the treatment might still be effective in the long term and supporting its recent accelerated approval for fALS treatment by the Food and Drug Administration (25 April 2023. It would be of outstanding importance to determine whether the treatment actually ablated mutant *SOD1* expression in astrocytes or any other cell type.

Among the different mechanisms mediating astrocyte toxicity towards neurons, functional impairment of the astrocyte plasmalemmal glutamate transporter EAAT2 has gained considerable interest as a potential therapeutic target from the milestone discovery that the antibiotic ceftriaxone enhances its expression and ameliorates the phenotype in mouse models of ALS [197] and FTLD-tau [156]. In spite of such promising pre-clinical results, ceftriaxone administration neither delayed the disease progression nor extended the survival of ALS patients in a phase III clinical trial [198]. Notably, a recent review of the literature discussed the extensive pre-clinical testing of ceftriaxone in the context of several neurological conditions and proposed that the drug should be administered prior to symptom onset to fulfil its therapeutic potential [199]. An alternative explanation for the failure of the clinical trial could be that ceftriaxone should be formulated to restrict its delivery to astrocytes to achieve the optimal effect on EAAT2 expression. Finally, the recent conflicting evidence that astrocytes expressing *CHMP2B* variants rather increase glutamate uptake and metabolism [157] suggests that not every patient might profit from EAAT2 overexpression, further highlighting the need for patient stratification and personalized medicine.

Another signaling pathway that raised considerable interest as a potential therapeutic target within astrocytes is the antioxidant response mediated by the transcription factor nuclear factor erythroid 2-related factor 2 (NRF2) [200]. The rationale for this choice was based on the overwhelming amount of evidence implicating defective NRF2 signaling in ALS patients [201,202] as well as in a wide variety of cell and animal models of the ALS/FTD spectrum [142,180,203,204,205,206,207,208,209,210,211]. Furthermore, pre-clinical studies convincingly showed that the selective rescue of functional deficit of astrocyte NRF2 is neuroprotective both in vitro [142,212,213,214] and in animal models [215], thus setting a strong rationale for astrocyte-targeted therapeutics. However, in spite of major progress in preclinical settings, specific targeting of astrocytes is yet to be implemented in the clinical practice [216]. Consistently, currently ongoing clinical trials are not investigating formulations to deliver NRF2-pathway-stimulating compounds specifically to astrocytes but rather exploring the therapeutic potential of nutritional supplementations with such biological action to the entire CNS. More specifically, studies have been registered aiming at evaluating whether supplementation with curcumin (found in turmeric), a polyphenol that stimulates the NRF2 signaling pathway [217], could delay progression in ALS patients (NCT04499963; NCT04654689). Although there are no current studies aiming at recruiting patients with FTD, it would be of interest to test the impact of curcumin also on individuals affected by this type of dementia. We envision that the subgroup of patients with FTLD-tau might gain the highest profit from supplementation with curcumin since this phenolic compound also binds to tau fibrils [218]. We can, therefore, speculate that it might interfere with inclusion body formation as well as enhance the anti-oxidant defense response.

In the previous section, we discussed how restoring the metabolic balance in ALS/FTD astrocytes might halt neuronal death. Intriguingly, the results of a pilot clinical trial (NCT02288091) showing the safety of inosine supplementation to ALS patients have been recently published [219]. Although the rationale for such study was to elevate blood concentration of uric acid, a known antioxidant whose levels correlate with slower progression rate and extended survival of ALS patients [220], we know now that inosine can also prevent astrocyte-derived motoneuron demise [96]. Therefore, we can speculate that any potential therapeutic benefit arising from inosine supplementation might be mediated also by rescuing astrocyte metabolic dysfunction. Similar considerations could be drawn about the compound CuATSM. The exact mechanisms of action of this drug are not yet known. Regardless, there are several clinical trials aiming at investigating its potential therapeutic effect in ALS (NCT04082832, NCT03136809, NCT04313166); these trials are perhaps prompted by CuATSM efficacy in ALS mouse models [221,222,223,224].

## 7. Conclusions

**Figure 3:**
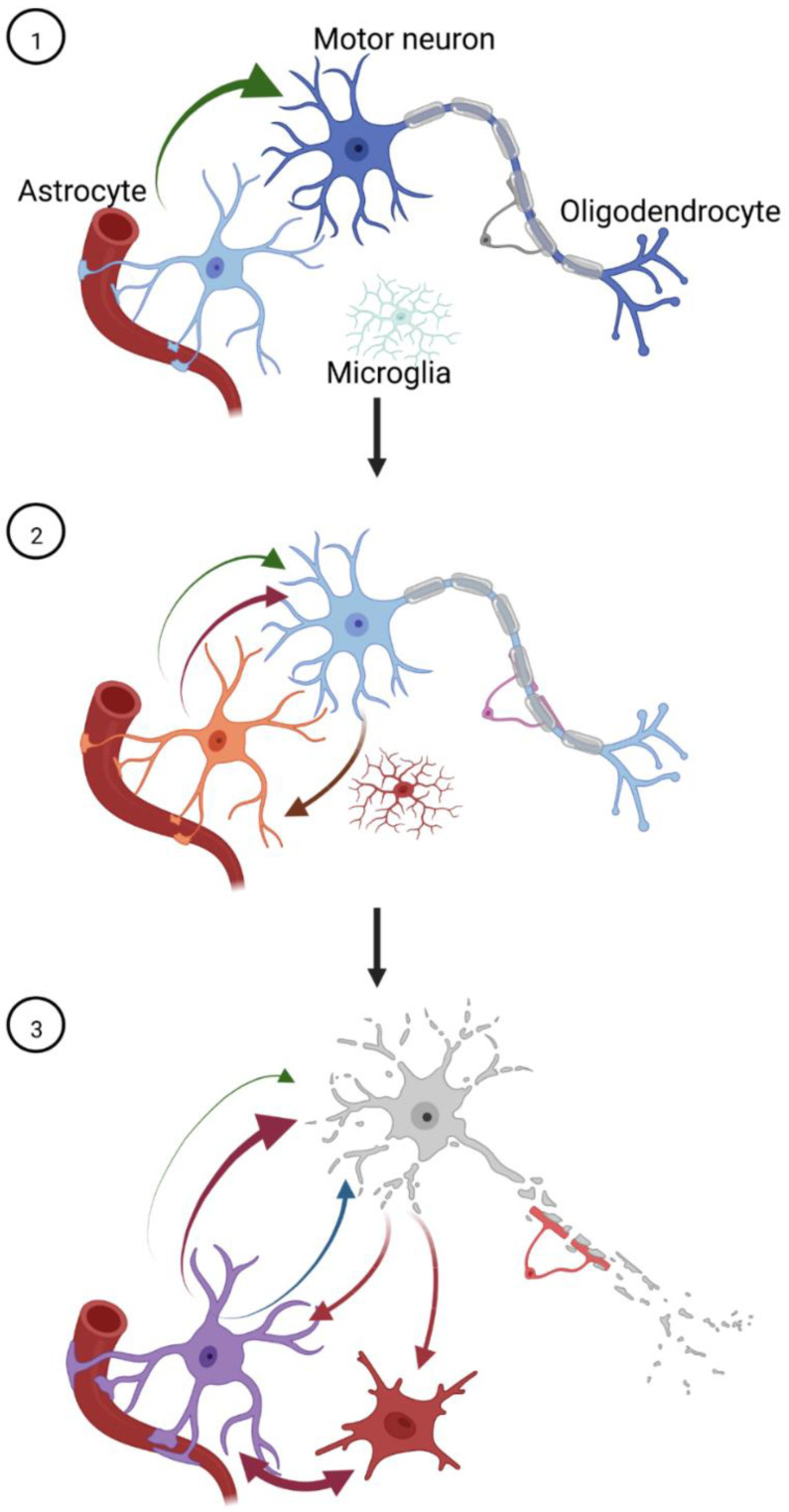
Hypothetical stage-dependent involvement of astrocytes in the pathogenesis of ALS and FTD/FTLD. Under physiological conditions (1), astrocytes engage in homeostatic signaling with neurons (thick green arrow) and other glial cell types to maintain the optimal milieu within the CNS. The expression of ALS/FTD-mutant proteins (2) or other environmental insults are sufficient to trigger an inflammatory response in astrocytes, diverting them from their housekeeping role (medium green arrow) towards a potentially harmful mutual interplay with neurons (red arrows). As neurodegeneration settles in (3), astrocytes are likely to acquire a full-blown neuroinflammatory phenotype that exerts further neurotoxic signaling, in cooperation with other glial cells (red arrows), compensatory neuroprotective messengers (blue arrow), and reduced trophic support (thin green arrow). Figure generated with Biorender.com.

In this review, we have extensively discussed considerable evidence demonstrating that astrocytes play a major role in the pathogenesis of ALS and FTLD. These observations suggest that any therapeutic regimen designed to halt neurodegeneration should include agents rescuing glial dysfunctions. Along with pharmacological interventions, rehabilitative training should be also considered to favor morphological remodeling and to improve the functional performance of astrocytes. Physical exercise [225,226,227] and environmental enrichment [225,228,229] were indeed reported to importantly reduce reactive astrocytosis and to alleviate the neuroinflammatory response in various animal models of injury and disease. In addition, several studies indicated that the expression of ALS- or FTD-associated mutant genes was sufficient to induce deleterious cell-autonomous changes in astrocytes as well as in other cell types of the CNS. During disease progression, neurodegeneration and microgliosis definitely increase the reactive status of astrocytes, thus intensifying several pathways, some of which can amplify the toxic environment, while others attempt to compensate by dampening neuronal injury (Figure 3). On the basis of these assumptions, we can conclude that a comprehensive understanding of the distinct responses of astrocytes requires urgent attention, with a particular focus on the mechanism(s) by which they integrate the different triggers of the disease (i.e., genetic or sporadic) as well as inter-individual genetic variability and personal habits. This information would hopefully guide the development of personalized treatments tailored to maximize the benefit for the different stages of the disease and patient individuality.

## Data Availability

Not applicable.
